# Refinement of recto-sigmoid colon vaginoplasty using a three-dimensional laparoscopic technique

**DOI:** 10.1097/MD.0000000000027042

**Published:** 2021-09-03

**Authors:** Jeong-ki Kim, Woong Na, Jeong Hyun Cho, Eun Jung Ahn, Eunyoung Kim, In-Gyu Song, Eon Chul Han, Dong Woon Lee, Byung Kwan Park, Yong-Gum Park, Beom Gyu Kim

**Affiliations:** a Department of Surgery, National Medical Center, Seoul, Republic of Korea; b Chung-Ang University College of Medicine, Seoul, Republic of Korea; c Department of Urology, National Medical Center, Seoul, Republic of Korea; d Department of Surgery, Dongnam Institute of Radiological and Medical Sciences, Busan, Republic of Korea; e Center for Colorectal Cancer, Research Institute and Hospital, National Cancer Center, Goyang, Republic of Korea; f Department of Surgery, Chung-Ang University College of Medicine, Seoul, Republic of Korea.

**Keywords:** neovagina, recto-sigmoid colon vaginoplasty, three-dimensional laparoscopy

## Abstract

To investigate the feasibility, safety, and outcomes of three-dimensional (3D) laparoscopic vaginoplasty with a rectosigmoid colon flap for vaginal reconstruction.

Following appropriate preoperative patient counseling, 17 consecutive patients underwent vaginoplasty using a 3D laparoscopic system. Perioperative and postoperative outcomes were retrospectively evaluated.

Between September 2016 and February 2020, 17 patients underwent 3D laparoscopic vaginoplasty with a rectosigmoid colon flap. Of them, 15 (88%) were transgender female patients, and 2 (12%) were cisgender female patients with congenital deformities. Among the 15 transgender patients, 12 (80%) underwent de novo surgeries and 3 (20%) underwent re-do surgeries. The mean age at the time of operation was 33.0 years, and the mean total operation time was 529 ± 128 minutes. The initial intraoperative mean vaginal depth was 15.2 ± 1.3 cm, and the 30-day readmission rate was 5.9% (1/17 cases). The mean follow-up duration was 24.8 months.

Perioperative and postoperative outcomes suggest that 3D laparoscopic rectosigmoid colon vaginoplasty is a potentially acceptable, effective, and safe method for vaginal reconstruction.

## 1 Introduction

Vaginal reconstruction is a procedure that is often performed on cisgender females with congenital vaginal absence and transgender women with a gender identity disorder. In cases of cisgender women with conditions such as Mayer-Rokitansky-Küster-Hauser (MRKH) syndrome, common reasons for surgical reconstruction include congenital adrenal hyperplasia, androgen insensitivity syndrome, gonadal dysgenesis, pelvic tumor, and trauma.^[1–4]^ The benefits of surgery include not only the creation of a vagina but also an improvement in the quality of life, psychological health, and sexual well-being of patients. Vaginal reconstruction is complex and requires a multidisciplinary team approach involving surgeons, psychiatrists, and endocrinologists.

Currently, vaginoplasty includes nonsurgical and surgical methods. Self-dilation therapy, including a single peritoneal flap in the creation of a neovagina, and self-vaginal dilation are currently the recommended non-surgical treatment options in patients with sexual development disorders.^[5,6]^ However, these take a toll on the patient's endurance in the long run and result in limited vaginal depth. Surgical methods for neovaginal reconstruction include penile inversion vaginoplasty, with or without additional skin grafts or flaps,^[5,6]^ peritoneal vaginoplasty, and intestinal vaginoplasty.^[7,8]^ The intestinal segment is isolated and transferred to the pelvic floor for vaginal reconstruction. In 1904, Baldwin first reported the use of the small intestine in vaginoplasty.^[9]^ Zangl and Pratt described the use of the sigmoid colon in vaginoplasty in 1961.^[10]^ Recently, the choices of donor sites have transitioned to the sigmoid colon from the small intestine. The advantages of sigmoid colon vaginoplasty include adequate vaginal width and depth, mucus secretion for lubrication, and facilitated sexual intercourse without postoperative shrinkage.^[7,11–17]^ Laparoscopy applications in this field have reported excellent visualization, minimal invasiveness, cosmetic outcomes, early recovery, and shorter hospital stay.^[18,19]^ Since only a limited number of studies have been reported in our country in this regard, we evaluated the technical feasibility and surgical outcomes of rectosigmoid colon vaginoplasty using a three-dimensional (3D) laparoscopy technique.

## 2 Materials and methods

### 2.1 Patients cohort

Between September 2016 and February 2020, a total of 17 patients underwent laparoscopic vaginoplasty with a rectosigmoid colon flap. Preoperatively, all patients underwent appropriate medical, psychological, and hormonal evaluations. The World Professional Association for Transgender Health Standards of Care were followed with respect to transgender patients.^[20]^ Informed consent was obtained from all patients.

### 2.2 Data analysis

We retrospectively reviewed the medical records of patients. The clinical characteristics of the patients were evaluated, including age, sex, body mass index, medical history, operation history, and intraoperative outcomes; total, laparoscopic, and perineal operation time; estimated blood loss; intraoperative event; postoperative outcomes; complications; Foley catheter removal; and length of hospital stay.

### 2.3 Preoperative management

Preparation for the operation included a semiliquid diet and bowel preparation with polyethylene glycol electrolyte lavage (Colyte, Taejoon, Seoul, South Korea) and bisacodyl (Dulcolax, Boehringer Ingelheim Pharmaceuticals, Ridgefield, CT, USA). We evaluated the condition of the sigmoid colon using a colonoscopic fiberscope before the operation. Polyps were found in 2 patients and were removed. Glycerin enemas were administered twice before surgery. Prophylactic intravenous antibiotics (second-generation cephalosporin) were administered 1 hour before the operation.

### 2.4 Technical considerations

All procedures were performed by 2 surgical teams, namely, laparoscopic and genital reconstructive surgeons. After the preparation of the perineal space by the genital surgery team, the laparoscopic surgery team joined the colon flap preparation stage. Laparoscopic engagement consisted of 4 steps: patient positioning and port placement, interposition of the rectosigmoid colon flap, creation of a space for the neovaginal canal, and anastomosis of the rectosigmoid flap to the distal part of the neovagina.

### 2.5 Patient positioning and port placement

All operations were conducted with the patients under general anesthesia placed in lithotomy positions on the operating table, which provided both abdominal and perineal exposure. A Foley catheter was inserted into the bladder. A laparoscopic surgeon operated using 5 small incisions (one 12 mm subumbilical portion, one 12 mm in the right lower quadrant, lateral to the inferior epigastric vessels, one 5 mm right lateral portion at the umbilical level, and two 5 mm left lateral and lower abdominal parts). Patients were placed in a right-sided Trendelenburg lithotomy position. After creating a pneumoperitoneum (12 mm Hg), the intra-abdominal and pelvic cavities were carefully explored using 3D laparoscopy (ENDOEYE FLEX Deflectable Videoscope, Olympus Corp., Tokyo, Japan).

### 2.6 Interposition of the recto-sigmoid colon flap

**Figure 1:**
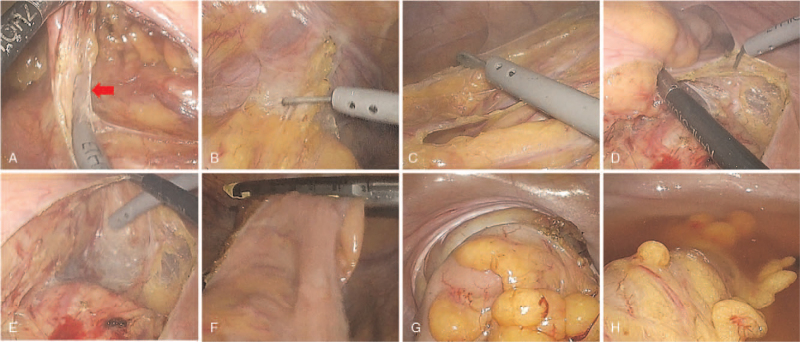
Laparoscopic procedure of recto-sigmoid colon flap preparation. (A): Dissection of the mesocolon including the inferior mesenteric artery. (B): Mobilization of the sigmoid colon from its lateral adhesion. (C): Mobilization of the descending colon. (D): Mobilization of the rectosigmoid colon. (E): Transmesorectal excision. (F): Distal resection of the rectosigmoid colon. (G): Intracorporeal end-to-end anastomosis. (H): Air leakage test at the anastomosis site. Red arrow: Inferior mesentery artery.

The interposition of the rectosigmoid colon flap is shown in Figure 1. The sigmoid colon was mobilized from its retroperitoneal attachments according to the principle of total mesorectal excision in rectal cancer surgery. Blunt dissection was used to lift the inferior mesenteric vessels away from the retroperitoneum and the presacral autonomic nerves. After selecting a rectosigmoid segment based on the vascular anatomy and mesenteric length, the mesosigmoid was released from its lateral adhesions, mobilized, and divided with an electrical energy device (LIGASure, Valleylab, Boulder, Colorado, USA) that uses ultrasonic energy for dissection, cutting, and coagulation. Once the sigmoid colon was released, the next step was to ensure the vascularization required for survival of the rectosigmoid segment. The rectum and sigmoid were dissected with an endoscopic linear cutter stapler (EndoGIA, Medtronic, Minneapolis, MN, USA) with a triple staggered row of staples through a 12-mm port in the right lower quadrant. The pedicled rectosigmoid colon was transected approximately 10 to 15 cm from the distal end and well-vascularized by the inferior sigmoid artery. A mini-laparotomy was performed at the subumbilical port site for extracorporeal proximal resection. Recto-sigmoid segment perfusion was checked by inspecting the mesenteric artery and its transverse pulsation. The distal end of the isolated rectosigmoid colon was sealed with a continuous locking suture and seromuscular interrupted suture to form the apex of the neovagina using sutures made from an absorbable material (3–0 Vicryl, Ethicon, Livingstone, UK) and transposed to the neovagina without tension on its vascular pedicle. End-to-end anastomosis (EEA) was performed between the colon and rectum to restore intestinal continuity, and the anvil of a curved intraluminal stapling device (EEA 31, Medtronic, Minneapolis, MN, USA) was inserted into the descending colon with a purse-string suture and introduced into the abdominal cavity. After recreating the pneumoperitoneum, intracorporeal end-to-end colorectal anastomosis was performed using an EEA 31 stapler through the anus and rectum. An air leakage test was conducted routinely by trans-anal insufflation of air after the bowel anastomosis site was immersed in warm saline.

### 2.7 Creation of space for the neovaginal canal

**Figure 2:**
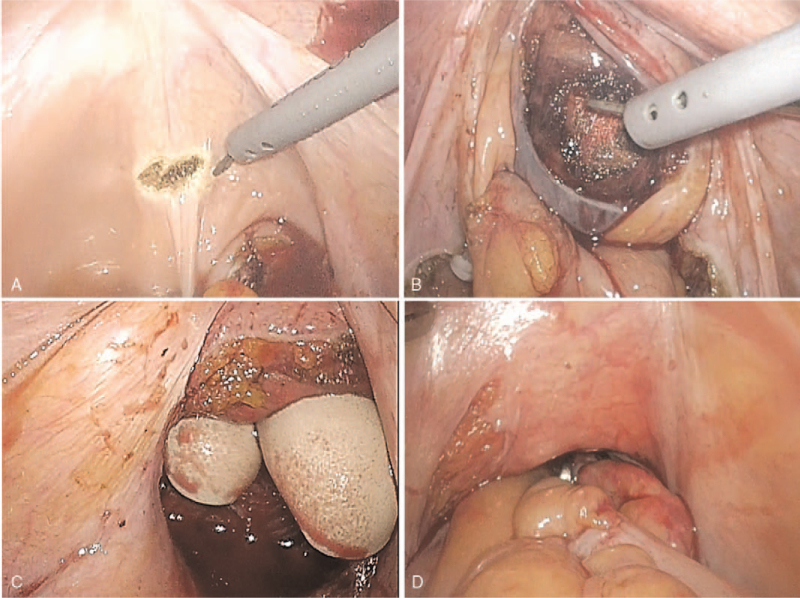
Laparoscopic procedure of creation of space for the neovaginal canal. (A): Dissection of the supravesical peritoneum. (B): Blunt dissection between the bladder and rectal space until the gauze is packed. (C): Measuring the perineal cavity from the caudal side with 2 fingers. (D): Interposition of the pedicled recto-sigmoid colon.

The creation of space for the neovaginal canal is shown in Figure 2. A key step in this procedure is the dissection between the rectum and the prostate-bladder complex to develop a space for the neovagina. The perineal surgical team designed and elevated the penile and scrotal skin flaps during gender-affirming surgeries. After removal of the male external genital organs by penectomy and bilateral orchiectomies, and creation of the female genital organ entities, the neovaginal cavity was formed through a blunt finger dissection to the peritoneal fold between the Denonvilliers’ fascia and the rectal wall. The gauze was placed in the neovaginal cavity to guide the intraperitoneal dissection of the tunnel. Under laparoscopic assistance, the surgical plane was developed between the urethra, bladder, and rectum, reaching down to the pelvis to form a canal with a width corresponding to approximately 2 fingers. To prevent postoperative neovaginal prolapse, the pedicled rectosigmoid colon was interposed in the peristaltic direction, and its distal ends were fixed at the bilateral periosteum of the pelvic outlet and ventrally to the neo-urethral opening.

### 2.8 Suturing the recto-sigmoid flap onto the distal part of the neovagina

Prerequisites for colocutaneous anastomoses are penectomy, orchiectomies, clitoroplasty, urethrotomy, vestibuloplasty, and labiaplasty. After the approximation of the distal rectosigmoid colon to the perineal skin, colo-cutaneus anastomosis using interrupted absorbable sutures was performed using the perineal approach.

### 2.9 Postoperative management

Maintaining a frog leg position was recommended to all patients for more than 3 days postoperatively to prevent wound disruption and retain the vitality of the rectosigmoid colon. Patients fasted until the first flatus and were then fed liquids and a semifluid diet. Open wound dressing was performed on the perineal wound 2 days post-surgery. Before discharge, all patients were trained in self-irrigation of the neovagina using saline to prevent diversion colitis and foul odor. The Penrose drainage tube at the genital wounds and urethral catheter indwelling were maintained for 2 weeks to prevent perineal wound disruption caused by the accumulation of edematous fluids and hematomes. Patients were encouraged to start using a vaginal dilator to prevent stenosis at the colo-cutaneous junction, as well as for pelvic floor muscle rehabilitation. They were allowed to resume sexual intercourse at 6 to 8 weeks post-surgery.

### 2.10 Statistical analysis

All statistical analyzes were performed using the software package SPSS version 26 (IBM Corporation, Armonk, NY). Descriptive statistics were used to analyze the data. Continuous variables are shown as mean ± standard deviation (SD), representing data with a normal distribution. Categorical variables are presented as percentages and/or numbers. Ethical approval for this study was obtained from the institutional ethics committee.

## 3 Results

Seventeen patients underwent colon vaginoplasty at our institution. The mean age at the time of operation was 33.0 years, and the mean body mass index was 23.7 ± 4.3 kg/m^2^ (Table 1). Of these, 15 cases (88%) were transgender female patients and 2 cases (12%) were cisgender female patients with congenital deformities such as MRKH syndrome and androgen insensitivity syndrome. Four patients had previously undergone abdominal surgery for appendicitis, traumatic splenic injury, and rectal injury during primary gender-affirming surgery. Rectal injury led to colostomies, colostomy repair, vaginal cavity creation failure, and crippled perineal structures (Table 2).

**Table 1:** Patient characteristics and summary of postoperative complications.

Patients (N = 17)	Mean ± SD (range)	Outcomes	N (%)
Age, yr	33.0 ± 11.8 (21–58)	Blood transfusion	4 (23.6)
BMI, kg/m^2^	23.7 ± 4.3 (19.7–32.7)	Colo-cutaneous junction annular stenosis	7 (41.1)
Operation Time, min	529 ± 128 (298–669)	30-day readmission	1
Laparoscopic surgery	192 ± 54 (140–370)	Urinary tract infection	1
Perineal surgery	337 ± 125 (98–482)	Delayed wound healing	1 (5.9)
Vaginal depth, cm	15.2 ± 1.3 (12.5–16)	Rectovaginal fistula	0
Estimated blood loss, mL	435.2 ± 214.8 (200–1000)	Anastomotic leakage	0
Mean first flatus, d	3.4 ± 1.33 (1–6)	Bowel obstruction	0
Mean length of stay, d	10.7 ± 1.7 (8–16)	Venous thromboembolism	0
Mean follow-up, d	743.7 ± 353.2 (62–1348)		

BMI = body mass index, d = day, SD = standard deviation.

**Table 2:** Summary of indication, operation type, operation time, complication, and follow-up.

#	Age	BMI (height /wt)	Medical and surgical history	Op-time, min	Transfusion	Complications	SA	FU (day)
Transgender de novo surgery								
1	29	20.6 (161.9/54.2)	Human immunodeficiency virus (HIV), Fistulectomy	547 (160 + 387)	−	UTI	A	1004
2	22	20.9 (174.5/63.9)		580 (190 + 390)	−	VS (3)	I	911
3	22	19.0 (166.8/53.1)	Mental retardation (MR), moderate	365 (160 + 205)	−		I	62
4	21	30.5 (163/81)		669 (190 + 479)	+	VS (18)	I	447
5	25	20.32 (166/55.9)	Treated cervical spine injury	628 (245 + 383)	−	VS (12)	A	704
6	22	20.4 (170.2/58.7)		637 (155 + 482)	+	VS (3)	A	107
7	31	24.0 (166/65.5)	Schizophrenia, controlled MR	490 (140 + 350)	−		I	315
8	30	32.7 (164.6/89.3)	Orchiectomy, Appendectomy,	656 (210 + 446)	+	VS (3)	A	84
9	47	19.7 (175.7/61.3)	HIV, poly-methyl-methyl-acrylamide hydrogel (PMMA) injection on genital skin	645 (180 + 465)	−	Wound problem	A	63
10	30	30.1 (167.1/83.5)		580 (170 + 410)	+	VS (3)	A	131
11	25	21.0 (168.9/60.2)	Orchiectomy	607 (185 + 422)	−		I	83
12	45	30.1 (167.6/85)		590 (165 + 425)	-	VS (3)	I	110
Transgender re-do Surgery								
13	37	23.9 (176.2/74.3)	Colostomy d/t rectal injury, 3 times of peritoneal surgery	614 (370 + 244)	−	VS (6)	A	1138
14	58	21.5 (167/59.3)	Skin vaginoplasty, Hyperlipidemia, Appendectomy	397 (165 + 172)	−		A	323
15	56	22.3 (164/59.5)	HIV, Skin vaginoplasty, spleen injury	455 (240 + 215)	-		A	274
Cisgender								
16	34	24.3 (166.4/67.1)	Mayer-Rokitansky-Küster-Hauser (MRKH) syndrome Salphingo-oophrorectomy, left	311 (150 + 161)	-		A	411
17	27	21.8 (165.8/60.2)	Androgen insensitivity syndrome Ovotestes removal	298 (200 + 98)	-		A	578

A = active, BMI (height/wt) = Body mass index (height in cm/ weight in kg), FU = follow up period (days), HIV = human immunodeficiency virus, I = inactive, MR = mental retardation, MRKH syndrome = Mayer-Rokitansky-Küster-Hauser syndrome, Op-time, min = Total operational time, min (laparoscopic + Perineal), PMMA = poly-methyl-methyl-acrylamide hydrogel, SA = sexual activity, UTI = urinary tract infection, VS = vaginal stenosis at the introital colo-cutaneous junction (sustained duration in months).

**Figure 3:**
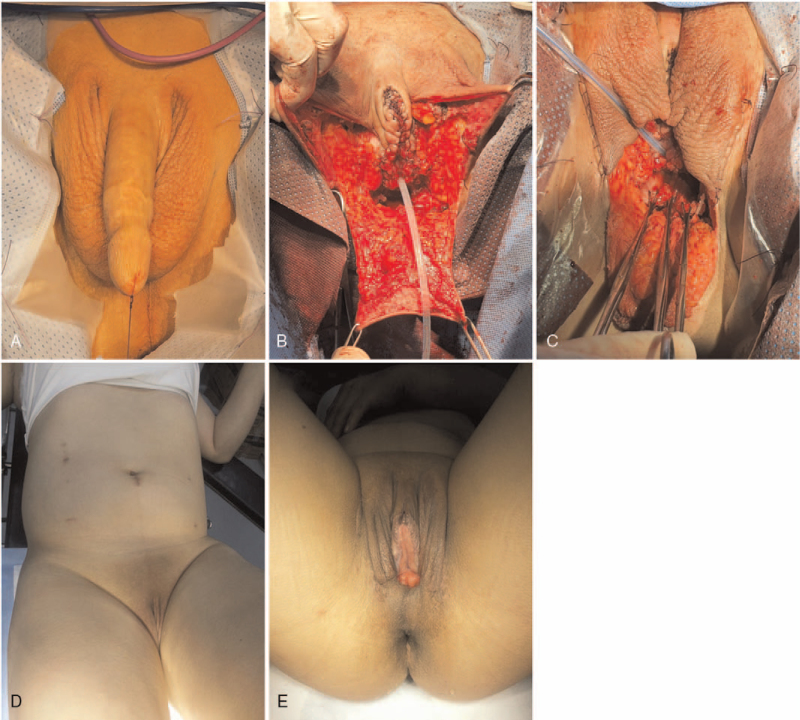
De novo gender affirming surgery; pictures of a 45-year-old male-to-female patient. (A): Preoperative view. (B): The midscrotal flap is prepared at the dorsal side for vaginal introitus after orchiectomies, clitoroplasty, vestibuloplasty, and perineal dissection. (C): Distal side of the colon flap is transferred through the perineal space using Babcock clamps; the left side of the flap is marked by a vessel clamp. (D, E): Abdominal and perineal status after 45 days.

**Figure 4:**
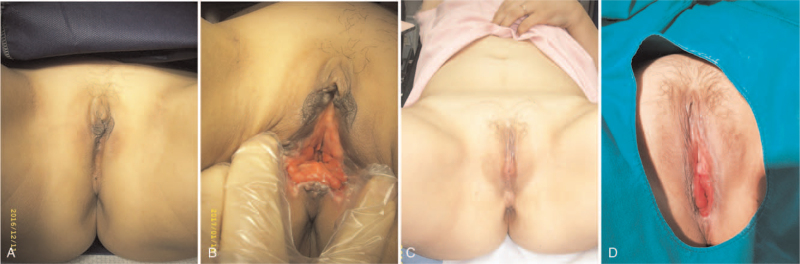
Disorder of sexual differentiation with vaginal dysgenesis. (A, B): Pre- and postoperative views of a 34-year-old patient with Mayer-Rokitansky-Küster-Hauser syndrome. (C, D): Pre- and postoperative views of a 27-year-old patient with androgen insensitivity syndrome.

Among the 15 transgender operations, 12 (80%) were de novo surgeries and 3 cases (20%) were re-do surgeries. Patients who previously did not undergo vaginoplasty underwent de novo surgery (Fig. 3). In de novo surgery, a mid-scrotal flap was prepared for the dorsal side of the vaginal introitus after orchiectomies, clitoroplasty, vestibuloplasty, and perineal dissection (Fig. 3B). After the approximation of the distal rectosigmoid colon to the perineal skin, a colocutaneous anastomosis was performed through the perineal approach (Fig. 3C). Two candidates for re-do surgery, who had developed a spacious vaginal introit width, wanted to resolve their concerns related to vaginal depth. In cisgender female patients with vaginal dysgenesis, creation of the peritoneal space was relatively straightforward because the underdeveloped vaginal vaults were directly in contact with the pelvic peritoneum (Fig. 4). After approximation of the distal rectosigmoid colon through the surgical opening at the dome of the vagina, the colovaginal anastomosis was performed through a perineal approach (Fig. 4B, D). The surgical procedures were performed successfully, and conversion to laparotomy was not required in any patient.

The mean total operative time was 529 ± 128 minutes. The mean laparoscopic surgery time was 192 ± 54 minutes, and the perineal surgery time was 337 ± 125 minutes. The mean length of the neovagina during surgery was 15.2 ± 1.3 cm. There were no intraoperative mortalities or complications. The mean total length of hospital stay was 10.7 ± 1.7 days. The postoperative course was uneventful, and the patients were usually discharged on postoperative day 8.7 ± 1.7. No complications occurred during the hospital stay. One patient reported delayed genital wound healing caused by a foreign body injection on the penile skin. Fatal surgery complications of colon vaginoplasty, such as anastomotic leakage and rectovaginal fistula, have not been reported. One patient (5.8%) experienced a minor complication whereby they developed a urinary tract infection (pyelonephritis) after discharge (Table 1).

A common concern after discharge from the hospital is annular stenosis at the colo-cutaneous junction, which is circular or partial, but not permanent. Regarding the transgender de novo surgery cases, 7 patients (68%; 7/12) suffered from this problem between 3 and 18 months. Regarding the re-do surgery cases, no one patient could undergo surgery due to rectal injury, and the remaining 2 patients with matured vaginal cavities did not experience annular stenosis at the colo-cutaneous junction. No neovaginal stenosis was found in the cisgender patients and those who did not have perineal tension caused by the prostate-seminal vesicle complex and pelvic floor muscles.

Positive sexual activity was reported in 65% of patients (11/17). In total, 50% (6/12) of the transgender patients who underwent de novo surgery and all cisgender and transgender patients who underwent re-do surgery reported positive sexual activity.

## 4 Discussion

Gender dysphoria, a synonym for gender incongruence, refers to the distress condition caused by a discrepancy between gender identity and the sex assigned at birth. Estimates of the proportion of transgender individuals within populations across the world ranged between 0.1% and 1.1% in reproductive-age adults.^[21]^ A survey of university students reported that gender dysphoria had an incidence of up to 0.8% in Japan^[22]^; however, the incidence and distribution of gender dysphoria in South Korea has rarely been reported. Recently, there has been an increase in the number of individuals with gender dysphoria seeking gender-affirming genital reconstruction.^[20,23,24]^ Vaginoplasty, the last step of the feminization process, involves the reconstruction of a neovagina that resembles a biological vagina in shape and function. The main goals of vaginoplasty are to achieve an esthetically and functionally ideal perineogenital complex that will satisfy the patient. In this study, we confirmed that 3D laparoscopic rectosigmoid colon vaginoplasty is a potentially acceptable, effective, and safe method for vaginal reconstruction.

Various vaginoplasty methods have been reported to have beneficial and limited outcomes. Vaginoplasty is ideally expected to be a safe procedure capable of providing adequate vaginal width and depth with minimal complications, quick recovery time, cosmetic benefits, and satisfactory sexual function. The Franks method is the most common non-surgical technique used to create a vagina. However, it requires long–term catheterization, and its functional success rate varies from 43% to 86%.^[25–28]^ Among the various surgical techniques, the most widely performed is the one in which a split-thickness skin graft covers a stent inserted into a surgically created space between the rectum and bladder.^[29]^ However, maintenance of the neovagina requires the daily application of a dilator for at least 6 months before sexual intercourse is possible. Such neovaginas commonly result in stenosis and shortening.^[30]^ Intestinal vaginoplasty has become a technique for vaginal reconstruction. Intestinal tissue proves sufficient neovaginal depth and lubrication and does not tend to shrink. However, the small intestine is too narrow for this purpose. Besides, it is difficult to bring the small intestine down to the pelvic cavity because of the short mesentery and long distance. The walls of the small intestine are relatively weak, and the mucosa is delicate and easily damaged with subsequent bleeding. Recto-sigmoid colon vaginoplasty, due to its proximity to the pelvic cavity at which it is to be used, is likely to be more suitable. The sigmoid colon offers an adequate length and natural lubrication of the neovagina.^[3]^ It also allows early coitus without the need for prolonged vaginal molding and self-dilation.^[13]^ Other advantages are the lack of shrinkage, narrowing, and stenosis at the perineal introitus.^[31]^ Furthermore, the thickness of the sigmoid wall seems to endure trauma better than the small intestine and skin grafts. Moreover, the proximity and easy mobilization of the vascular pedicle ensures that serious complications, such as segmental necrosis and fistula formation, rarely occur. An overview of the literature on surgical complications after laparoscopic sigmoid colon vaginoplasty is presented in Table 3.^[32–36]^ Some intra- or postoperative complications, including anastomotic leakage, anastomotic stenosis, intra-abdominal bleeding, rectal perforation, bladder injury, adhesive ileus, and introital stenosis, are caused by the use of the intestinal part or intestinal tissue as a vaginal lining during vaginoplasty (Table 3). However, these complications rarely occurred during the 3D vaginoplasty procedure using colon tissue, as seen in our data. Considering these results, we postulated that 3D laparoscopic surgery can be applied to vaginoplasty using colon tissue as a safe and feasible technique.

**Table 3:** Overview of the literature on surgical complications after laparoscopic sigmoid colon vaginoplasty.

Author, year	N	Patient characteristics	Primary or secondary	Complications, N (%)
Cai et al, 2007 ^[32]^	26	Cisgender women	Primary (26)	Introital stenosis, 2 (7.7) Bowel obstruction, 1 (3.8) Wound infection, 1 (3.8)
Cao et al, 2013 ^[33]^	14	Cisgender women	Primary (26)	Anastomotic leakage, 1 (7.1) Urinary tract infection, 1 (7.1) Hydronephrosis, 1 (1.7)
Van der Sluis et al, 2016 ^[34]^	21	Transgender women	Secondary (21)	Rectal perforation, 2 (9.5) Intraoperative bladder injury, 1 (4.8) Anastomotic stenosis, 1 (4.8) Bowel obstruction, 1 (4.8)
Bouman et al, 2016 ^[35]^	42	Transgender women	Primary (42)	Introital stenosis, 6 (14.3) Postoperative bleeding, 2 (4.7) Rectal perforation, 1 (2.3) Mucosal prolapse, 1 (2.3) Anastomotic leakage, 1 (2.3) Death due to necrotizing fasciitis, 1 (2.3)
Salgado et al, 2018 ^[36]^	12	Transgender women	Primary (12)	Bowel obstruction, 2 (16.7) Intraoperative bladder injury, 1 (8.3) Deep vein thrombosis, 1 (8.3) Pulmonary embolism, 1 (8.3) Wound infection, 1 (8.3)

Our study demonstrates that laparoscopic rectosigmoid colon vaginoplasty can be performed safely and that the short-term results are satisfactory. Laparoscopic procedures are cosmetically much more acceptable to young patients than scarring, which results from laparotomy or skin graft donor sites. As laparoscopic procedures are minimally invasive, they reduce the incidence of wound complications, postoperative pain, paralytic ileus, and adhesive complications of the intestine that permit the early recovery of patients.^[2,18,37]^ Serious complications such as postoperative anastomotic leakage or stenosis, rectovaginal fistula formation, colon flap necrosis, and necrotizing cellulitis are rare but can occur after colon vaginoplasty. Anastomotic leakage is reported in 0% to 7% of all cases after intestinal vaginoplasty.^[7]^ However, intraoperative and postoperative complications were rarely observed, and the incidence of re-hospitalization was very low. These results suggest that 3D laparoscopic rectosigmoid colon vaginoplasty is a safe and reliable technique for the creation of a neovagina.

To the best of our knowledge, no study has compared the surgical outcomes between two-dimensional (2D) and 3D vaginoplasty using colon tissue. In general, 3D laparoscopy overcomes the disadvantages of conventional 2D laparoscopy, providing surgeons with stereoscopic vision in which depth perception is achieved using different unique images received by each lens.^[38,39]^ 3D laparoscopy involves a dual-lens system in which 2 separate lenses are present within a single laparoscope along with 2 cameras. Compared with 2D laparoscopy, it is easier to precisely grasp and dissect tissue, control bleeding, and ligate blood vessels with 3D laparoscopy as it offers the surgeon improved depth perception, spatial location, and hand-eye coordination.^[40]^ Tanagho et al demonstrated that 3D visualization significantly enhanced the ease and efficiency of basic laparoscopic skills, and hastened the development of surgical proficiency.^[38]^ Sørensen et al reported that 3D laparoscopy resulted in improved operation times and reduction in the number of repetitions and performance errors compared to 2D laparoscopy.^[41]^ The laparoscopic technique allows a better view of the pelvic floor and recto-neovaginal space to avoid rectal injury. Genitourinary dysfunctions are well-known problems after sigmoid colon and rectal surgery and are caused by pelvic autonomic nerve damage. To preserve voiding and sexual function after pelvic surgery, it is important to identify the pelvic autonomic plexus and neurovascular bundles during pelvic dissection. Adequate vision, traction, and counter-traction are important factors for successful pelvic operation with good functional outcomes. 3D laparoscopy can facilitate the preservation of the pelvic autonomic nerves, thereby achieving favorable postoperative voiding and sexual function, as seen in this study. To safely perform laparoscopic colon vaginoplasty, an expert team with the presence of a colorectal surgeon with advanced laparoscopic skills is desirable. Bouman et al reported intraoperative rectal injury, anastomotic leakage, postoperative bleeding, and urinary retention during primary total laparoscopic sigmoid vaginoplasty in transgender women with penoscrotal hypoplasia^[34]^; however, these complications were not reported in the present study.

Few cases of adenocarcinoma in the sigmoid neovagina have been reported,^[42,43]^ but the overall prevalence is unknown. Theoretically, repetitive inflammation may lead to the risk of sigmoid neovaginal malignancies. There are no clear guidelines for cancer surveillance following colon vaginoplasty. We recommend checking the neovagina by performing routine colonoscopy for postoperative neovaginal cancer surveillance.

A limitation of this study is that it was a retrospective, single-center analysis with a small sample size and a relatively short follow-up duration.

## 5 Conclusion

This study demonstrated that 3D laparoscopic rectosigmoid colon vaginoplasty is a safe and feasible technique for the creation of a neovagina. Its benefits include the provision of good spatial vision to the surgeon, which can help reduce errors, especially during initial procedures, as well as reduce morbidity.

## Author contributions

**Conceptualization:** Jeongki Kim, Beom Gyu Kim.

**Data curation:** Woong Na, Jeong Hyun Cho.

**Formal analysis:** In-Gyu Song.

**Investigation:** Eun Jung Ann.

**Methodology:** Eunyoung Kim.

**Software:** Eon Chul Han.

**Supervision:** Yong-Gum Park, Byung Kwan Park.

**Visualization:** Dong Woon Lee.

**Writing – original draft:** Jeongki Kim, Beom Gyu Kim.

**Writing – review & editing:** Jeongki Kim, Woong Na, Jeong Hyun Cho, Eun Jung Ann, Eunyoung Kim, In-Gyu Song, Eon Chul Han, Dong Woon Lee, Byung Kwan Park, Yong-Gum Park, Beom Gyu Kim.
